# In Vitro Evaluation of Biological Activities and Phytochemical Analysis of Different Solvent Extracts of *Punica granatum* L. (Pomegranate) Peels

**DOI:** 10.3390/plants10122742

**Published:** 2021-12-13

**Authors:** Mohamed Taha Yassin, Ashraf Abdel-Fattah Mostafa, Abdulaziz Abdulrahman Al Askar

**Affiliations:** Botany and Microbiology Department, College of Science, King Saud University, P.O. Box 2455, Riyadh 11451, Saudi Arabia; aalaskara@ksu.edu.sa

**Keywords:** pomegranate, food spoilage, antibacterial, antiradical, carcinopreventive, gas chromatography-mass spectrometry

## Abstract

Antimicrobial resistance is a public health concern resulting in high rates of morbidity and mortality worldwide. Furthermore, a high incidence of food poisoning diseases besides harmful implications of applying synthetic food additives in food preservation necessitates fabrication of safe food preservatives. Additionally, damaging effects of free radicals on human health has been reported to be involved in the incidence of serious diseases, including cancer, diabetes and cardiovascular diseases; hence, finding safe sources of antioxidants is vital. Therefore, the present study was carried out to assess the antibacterial, antiradical and carcinopreventive efficacy of different solvent extracts of pomegranate peels. Agar disk diffusion assay revealed that *Staphylococcus aureus*, MRSA, *E. coli* and *S. typhimurium* were highly susceptible to methanolic fraction of *Punica granatum* L. peels recording inhibition zones of 23.7, 21.8, 15.6 and 14.7 mm respectively. Minimum inhibitory concentration (MIC) and minimum bactericidal concentration (MBC) of the methanolic fraction of *Punica granatum* L. peels against *S. aureus* were 0.125 and 0.250 mg/mL, respectively. In addition, the pomegranate acetonic and methanolic fractions revealed an impressive antiradical efficiency against DPPH (2,2-diphenyl-1-picrylhydrazyl) radical recording radical scavenging activity percentages of 86.9 and 79.4%, respectively. In this regard, the acetonic fraction of pomegranate peels revealed the highest anti-proliferative efficiency after 48 h incubation against MCF7 cancer cells recording IC_50_ of 8.15 µg/mL, while the methanolic extract was highly selective against transformed cancer cells compared to normal cell line recording selectivity index of 5.93. GC–MS results demonstrated that 5-Hydroxymethylfurfural was the main active component of methanolic and acetonic extracts of pomegranate peels recording relative percentages of 37.55 and 28.84% respectively. The study recommends application of pomegranate peel extracts in the biofabrication of safe food preservatives, antioxidants and carcinopreventive agents.

## 1. Introduction

Antimicrobial resistance represents a significant health threat to human health resulting in annual hospitalizations of 24 million and over 700,000 deaths annually [1]. Resistant bacterial pathogens are one of the crucial factors of disability and fatality worldwide, representing a global health burden [2]. Methicillin-resistant *Staphylococcus aureus* (MRSA) initially emerged in 1961, contributing to high rate of death and disability in both community and healthcare facilities [3]. 

Food poisoning diseases represents a global health burden, which is caused by ingestion of food products contaminated with pathogenic bacteria [4]. Contamination of food with pathogenic bacteria can occur at different stages of food processing, including harvesting, packaging and slaughtering [5]. The bacterial food spoilage microbes can withstand the unfavorable conditions of food preservation, such as vacuum packaging, low temperature and pasteurization [6]. In this regard, *Staphylococcus aureus* can remain viable for a long time outside the human body, especially in dried conditions [7]. *S. aureus* can cause food borne illness due to the ingestion of enterotoxin at the optimal temperature range of 40 to 45 °C resulting in food poisoning symptoms, such as headache, nausea, vomiting and general weakness [8]. *Escherichia coli* is a facultative anaerobic bacterial strain belonging to Gram-negative bacteria, which can be transmitted through consuming food infected with feces of animals or humans [9]. The pathogenic *E. coli* O157:H7 strain initially emerged in 1982 causing mild to severe diarrhea and resulting in high numbers of illnesses and hospitalizations every year [10]. *Salmonella* spp. are considered one of the main etiological agents, which cause food borne outbreaks resulting in 1.5 million infections and 26,500 hospital cases annually [11]. Regarding food preservation, the usage of synthetic food additives represents a health concern due to the deleterious impact of these additives on human health [12]. Hence, finding new and safe approaches for food preservation is urgently needed for combatting pathogenic microbes and increasing the food shelf-time avoiding use of synthetic chemical additives [13]. 

The free radicals are considered disease causing agents of severe diseases, such as cancer, cardiovascular diseases, diabetes, arteriosclerosis and stroke [14]. The oxidative stress disrupts the level of antioxidants in the biological system resulting in damage to various cellular constituents as DNA, carbohydrates, proteins and lipids [15]. Certain plant extracts have been reported as potential source of antioxidants due to their phytochemical ingredients of phenolics and flavonoids [16]. The phytochemicals of plant extracts have been employed as a potential source of natural antioxidants avoiding the detrimental effects of artificial antioxidants as possible toxicity and carcinogenesis [17]. The natural antioxidants act as a free radical scavenging agent by donating hydrogen and/or electrons resulting in minimizing the destructive effects of these radicals [18]. 

Cancer is considered the second main cause of mortality globally. The recorded mortalities due to cancer around the world are reported to be 35 million every year [19]. The development of cancer occurs due to disturbance in the cellular mechanisms responsible for apoptosis and cell division [20]. Breast cancer is considered one of the dominant cancers worldwide, contributing to a high mortality rate among women [21]. Globally, one woman among every eight women has the risk of invasive breast cancer progression during lifetime [22]. In terms of cancer therapy, the conventional chemotherapy is reported to cause severe side effects, such as vomiting, nausea, gastric ulcers and alopecia [23]. The phytochemicals of plant extracts were reported to acquire pro-apoptotic and anti-proliferative properties suggesting the use of these natural products as carcinopreventive adjuvant therapies [24]. Regarding the safety during cancer treatment, using natural products such as cancer adjuvant therapies is considered an impressive way for cancer treatment in evading the detrimental effects of chemotherapeutic drugs [25].

Owing to the high mortality and morbidity rate resulting from antimicrobial resistance and food poisoning diseases worldwide, finding new and safe approaches of anti-microbial agents are urgently needed. Moreover, the noxious impact of free radicals on an individual’s health besides the pernicious effects of synthetic antioxidants necessitates searching for new sources of safe and natural antioxidants. Additionally, finding new ways for cancer therapy instead of the conventional chemotherapy is urgently needed due to the severe side effect of chemotherapy on the human health. Hence, the present study was carried out to examine the antimicrobial, antiradical and anti-proliferative activities of pomegranate peel extracts as efficacious natural and secure therapeutic agents. 

*Punica granatum* L. (pomegranate) fruit belongs to the Punicaceae family and is distinguished by its high nutritive benefits assignable to their bioactive components of phenolic acids, flavonoids and tannins [26]. In this regard, pomegranate plant has been assayed for possible healing effects recording antiradical, antimicrobial, anti-inflammatory, hypolipidemic, antiproliferative and hypoglycemic properties [27]. The potential antiradical and antitumor efficiency of pomegranate peel extracts has been directly assigned to their phytoactive constituents of polyphenolic compounds [28].

*Punica granatum* L. peels were previously described to exhibit antimicrobial efficiency against food borne pathogens involving *Escherichia coli*, *Bacillus subtilis*, *Penicillium italicum* and *Fusarium sambucinum* [29]. Moreover, pomegranate exhibited strong antioxidant activity due to the prevalence of several active phytochemicals as polyphenols, flavones, flavonoids, anthocyanins and catechins in seeds, fruits and peels of pomegranate [30]. Furthermore, pomegranate has been reported as a potential source of anti-tumor agents owing to the prevalence of many active phytochemicals as polyphenols and flavonoids [31]. In this regard, recent a study evaluated the efficiency of Turkish pomegranate juice as an antiproliferative agent against MCF-7 human breast cancer line and demonstrated the potent cytotoxic effect of pomegranate juice on cancer cells recording IC_50_ of 49.08 µg/mL [32]. The previous literature focused on the evaluation of biological parameters of pomegranate seed extracts, thus the current study was conducted to assess the different biological properties of different solvent extracts of pomegranate peels as antibacterial, antioxidants and antitumor agents. Furthermore, the present study also evaluated the selectivity index and the hemolytic activity of the different fractions of pomegranate peels to ensure the biosafety of these extracts. 

## 2. Results

### 2.1. Extraction Yield

Methanol solvent exhibited the highest efficiency in extraction followed by acetone and hexane recording yield percentages of 9.82, 6.51 and 4.98%, respectively. 

### 2.2. Antibacterial Susceptibility Testing

**Figure 1:**
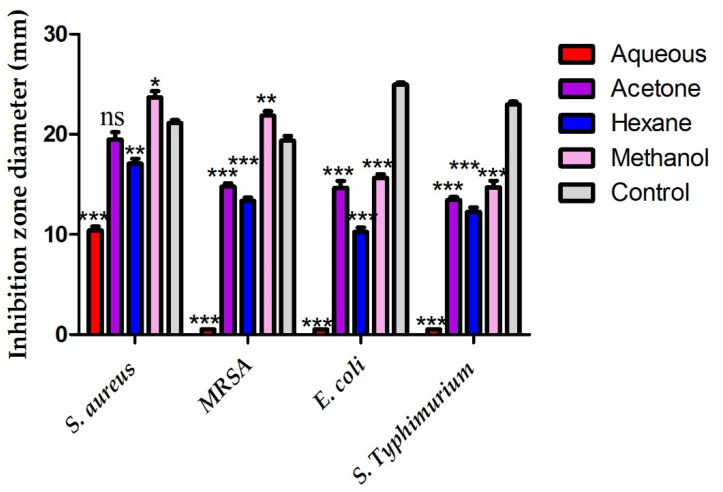
Antimicrobial activity of different pomegranate peel extracts against different bacterial pathogens. Asterisks indicated the significance difference between the extracts and control (*** *p* ≤ 0.001, ** *p* ≤ 0.01, * *p* ≤ 0.05, ns: non-significant).

The concerned bacterial strains showed different susceptibilities to the pomegranate peel extracts. The tested bacterial strains (*S. aureus*, MRSA, *E. coli* and *S. typhimurium*) were highly susceptible to the pomegranate methanolic extract recording suppressive zones of 23.7, 21.8, 15.6 and 14.7 mm, respectively. The antibacterial efficiency of the pomegranate methanolic extract against *S. aureus* and MRSA strains was significantly higher than that of the control (*p* ≤ 0.05). The aqueous extract of pomegranate peels showed antibacterial activity against S. aureus with inhibition zone diameter of 10.44 mm while no antibacterial activity was detected against MRSA, *E. coli* and *S. typhimurium*. The gram positive bacterial strains (*S. aureus* and MRSA) showed a superior susceptibility to pomegranate peel extracts in comparison with the gram negative bacterial strains (*E. coli* and *S. typhimurium*) as seen in Figure 1. 

### 2.3. Determination of Minimum Inhibitory Concentration (MIC) and Minimum Bactericidal Concentration (MBC)

MIC of the pomegranate methanolic extract against *S. aureus* was found to be 0.125 mg/mL confirming that *S. aureus* was the most sensitive strain to the pomegranate extracts. In addition, the methanolic extract suppressed the bacterial growth of the resistant MRSA strain at the concentration of 0.250 mg/mL while the cidal concentration was 0.5 mg/mL as showed in Table 1. The MIC of pomegranate extract against Gram negative bacterial strains was 0.5 mg/mL while the cidal concentrations were 1.00 and 2.00 mg/mL against *E. coli* and *S. typhimurium*, respectively.

### 2.4. Antiradical Efficiency of Pomegranate Extracts

**Figure 2:**
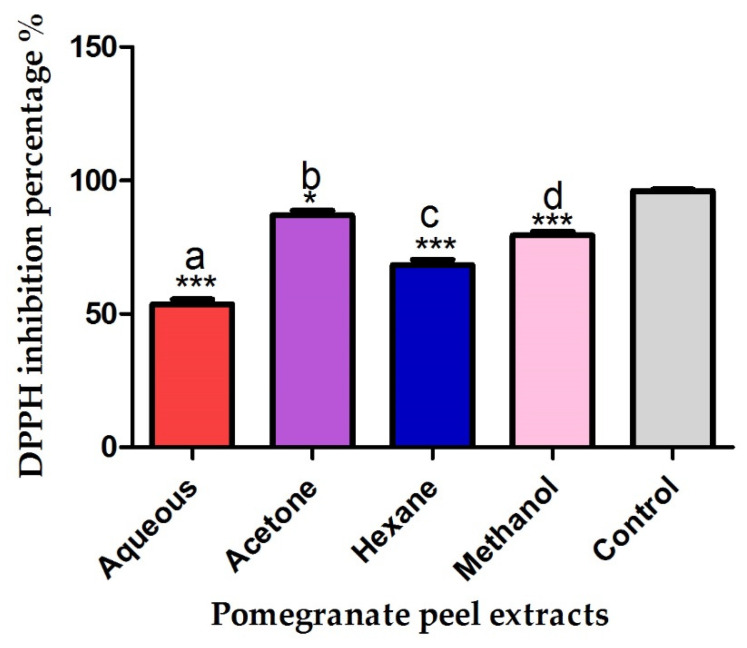
Antiradical efficiency of pomegranate peel extracts against DPPH radical. Different letters indicate that values were significantly different (*p* ≤ 0.05). Asterisks indicated the significance difference between the extracts and control (*** *p* ≤ 0.001, * *p* ≤ 0.05).

The suppressive effect of pomegranate peel extracts against DPPH radical was significantly variable among different extracts. The acetonic extract of pomegranate peels showed the highest antiradical activity followed by the pomegranate peel methanolic, hexanic and water extracts with relative percentages of 86.9, 79.4, 68.2 and 53.7% respectively as seen in Figure 2. The DPPH inhibition percentages of different pomegranate peel extracts were significantly different compared to control (*p* ≤ 0.05).

### 2.5. In Vitro Antiproliferative Assay

**Figure 3:**
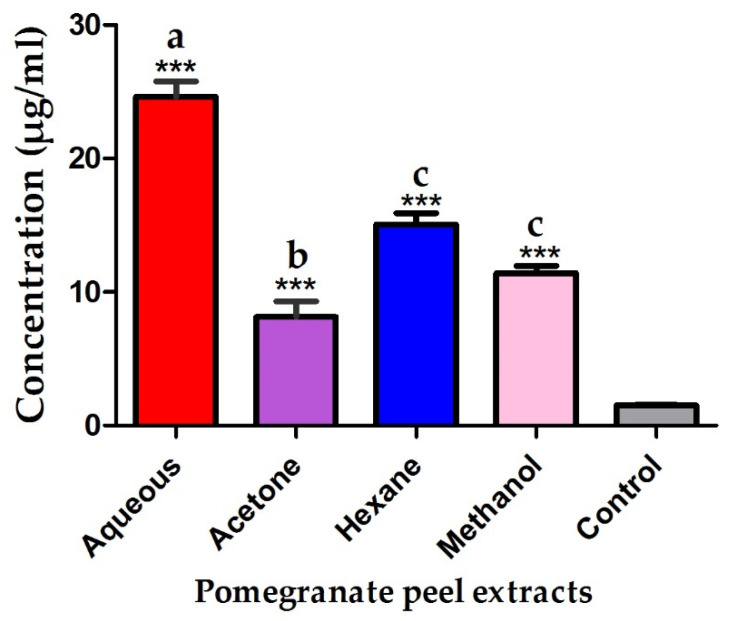
IC_50_ of pomegranate peel extracts against MCF7 cancer cells. Different letters indicate that values were significantly different (*p* ≤ 0.05). Asterisks indicated the significance difference between the pomegranate peel extracts and control (*** *p* ≤ 0.001).

**Figure 4:**
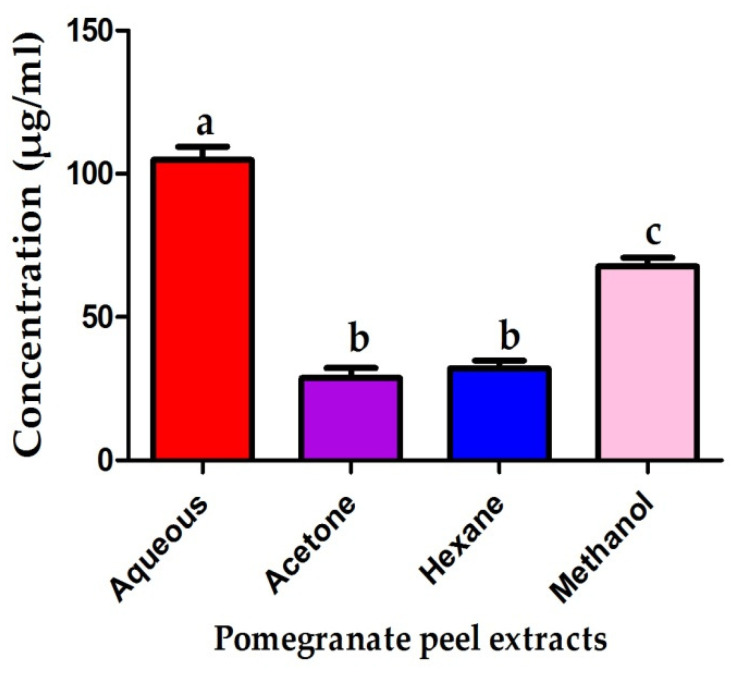
IC_50_ of pomegranate peel extracts against normal human fibroblasts (WI38). Different letters indicate that values were significantly different (*p* ≤ 0.05).

The antiproliferative efficiency of pomegranate peels against cancer cells was significantly variable among different extracts. The acetonic extract of pomegranate peels expressed the highest antiproliferative effect after 48 h incubation against cancer cells while the aqueous extract revealed the least potency with IC_50_ of 8.15 and 24.64 µg/mL respectively. Furthermore, the pomegranate peel hexanic and methanolic extracts showed a moderate antiproliferative activity against MCF7 cells with IC_50_ of 15.07 and 11.41 µg/mL as seen in Figure 3. Moreover, the antiproliferative efficiency of different pomegranate peel extracts was significantly different compared to Toxol chemotherapeutic agent (*** *p* ≤ 0.001). The antiproliferative activity of pomegranate peel extracts against normal human fibroblasts (WI38) was also evaluated to detect the selectivity index of the different extracts. The aqueous extract exhibited the lowest cytotoxic effect against normal human fibroblasts (WI38) while the acetonic extract revealed the highest toxicity with IC_50_ of 104.8 and 28.7 µg/mL respectively as shown in Figure 4. The methanolic extract was highly selective against malignant cancer cells while the hexanic extract exhibited the lowest selectivity recording selectivity indexes of 5.93 and 2.13, respectively. The acetonic and aqueous extracts of pomegranate peels revealed moderate selectivity against malignant cells recording selectivity indexes of 3.52 and 4.26, respectively. 

### 2.6. The Erythrocytes Hemolytic Assay

**Figure 5:**
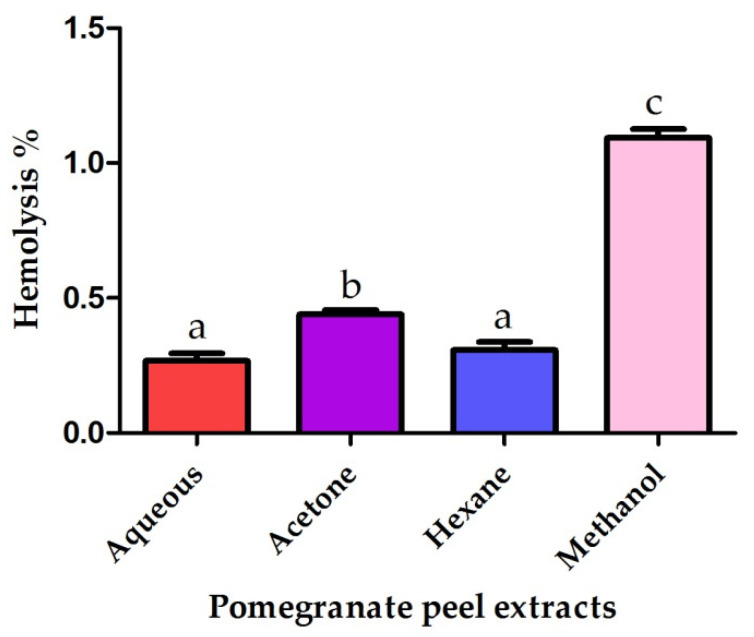
Cytotoxic effects of pomegranate peel extracts against human erythrocytes. Different letters indicate that values were significantly different (*p* ≤ 0.05).

The pomegranate peel extracts exhibited no hemolytic activity against human erythrocytes at a concentration of 10 mg/mL. The methanolic extract revealed the highest hemolytic activity, while the water extract showed the least hemolytic activity as seen in Figure 5. The hemolytic assay confirmed the safety of the application of pomegranate peel extracts as source of natural antioxidants and antiproliferative agents against cancer cells. 

### 2.7. Determination of Total Phenolic Content

**Figure 6:**
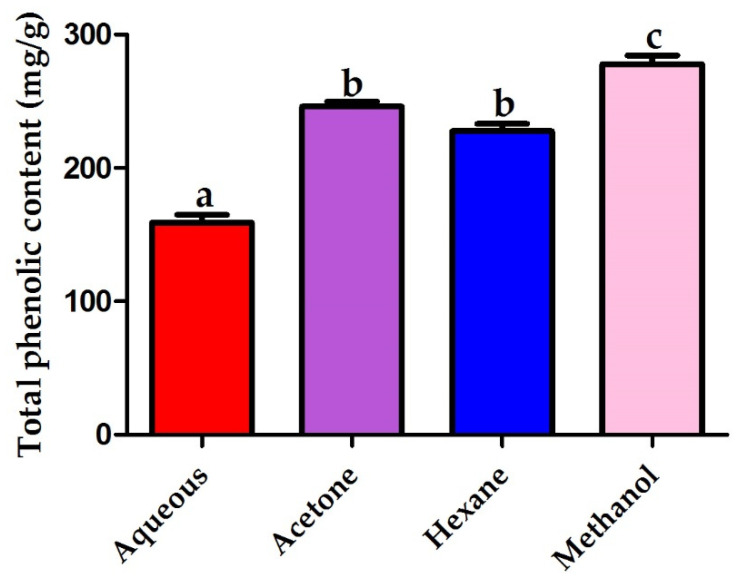
Total phenolic content (mg/g) of different solvent extracts of pomegranate peels. Different letters indicate that values were significantly different (*p* ≤ 0.05).

The methanolic extract of pomegranate peel revealed the highest polyphenolic content recording 277.8 mg gallic acid equivalents (GAE)/gram of the pomegranate extract. In contrast, the pomegranate aqueous extract recorded the lowest polyphenolic content of 159 mg GAE/g. The acetonic and hexanic extracts of pomegranate peels recorded polyphenolic content of 246 and 227.7 mg GAE/g, respectively as seen in Figure 6. 

### 2.8. GC–MS Analysis of Pomegranate Peel Extracts

**Figure 7:**
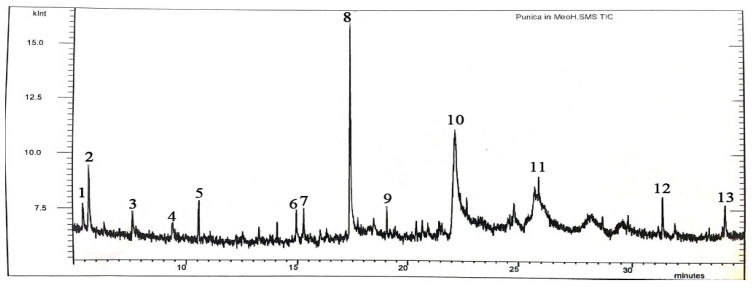
Chromatogram of the methanolic extract of pomegranate peel.

GC–MS investigation of the methanolic extract of pomegranate peel revealed that 5-Hydroxymethylfurfural (37.55) was the main active ingredient followed by Octadecanoic acid (16.89%), Furfural (14.62%), γ-Sitosterol (9.23%), Glycerin (7.74%), Heptasiloxane, hexadecamethyl- (3.14%), Pyrazole [4,5-b]imidazole, 1-formyl-3-ethyl-6-β-d-ribofuranosyl (2.39%), Lanosterol (1.82%), Cycloartenol acetate (1.64%), Cyclobutylamine (1.58%), Palmitic acid (1.18%), 4H-Pyran-4-one, 3,5-dihydroxy-2-methyl (1.14%) and L-Glucose (1.07%), respectively as shown in Table 2, Figure 7. Furthermore, the pomegranate peel acetonic extract was comprised of 5-hydroxymethylfurfural (28.41%) as main phytoactive compound, followed by Furfural (11.29%), 2-Furancarboxaldehyde, 5-methyl (9.58%), N-phenyl-2-naphthalenamine (8.47%), 2,5-Furandione, 3-methyl (7.12%), n-Hexadecanoic acid (6.85%), D-Arabinose (5.78%), Squalene (4.29%), 2-ethyl-1,3-dimethyl-benzene (4.25%), 4H-Pyran-4-one, 3,5-dihydroxy-2-methyl, (3.67%), Lanosterol (2.89%), 4-Methyl itaconate (2.45%), Hexadecanoic acid, methyl ester (1.89%), Eicosane (1.56%) and α-Cubebene (1.06%), respectively as seen in Table 3. On the other hand, The hexanic extract of pomegranate peel was mainly comprised of Hexasiloxan, tetradecamethy (38.28%) as a major active component followed by Aminopropionic acid (19.46%), Dicholoroacetamide (11.23%), 2,6-Di-tert-butylphenol(8.12%), Trioxsalen(8.12%), 2,6-Dimethyl-3,4-bis(trimethylsilyloxymethyl)pyridin(5.23%), Octadecanoicacid,2-propenylester(4.85%), Octadecenoate(2.78%), 4H-Pyran-4-one,2,3-dihydro-3,5-dihydroxy-6-methyl- (1.98%) and Benzeneacetic acid (1.28%), respectively as seen in Table 4. 

### 2.9. HPLC Analysis of Pomegranate Peel Extracts

**Figure 8:**
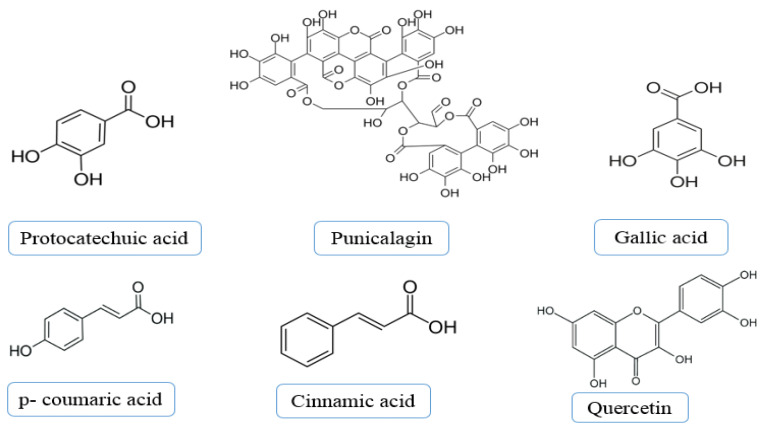
Phenolic compounds detected in the methanolic extract of pomegranate peel by HPLC analysis.

Identification and quantitative analyses of different phenolic compounds in methanolic extracts of pomegranate peels were performed using HPLC. The phenolic compounds in the pomegranate methanolic extract were investigated as the methanolic extract recorded the highest antibacterial activity and highly effective against transformed cell line. The phenolic compounds identified in the methanolic extract of pomegranate peel are shown in Figure 8. The HPLC analysis investigated the presence of six polyphenolic constituents including punicalagin, gallic acid, cinnamic acid, quercetin, protocatechuic acid and p-coumaric acid. Cinnamic acid was the main phenolic component (31.69%) followed by quercetin (20.78%), p-coumaric acid (19.85%), protocatechuic acid (10.78%), punicalagin (9.12%) and gallic acid (7.89%) as seen in Table 5.

## 3. Discussion

The tested bacterial strains showed a variable susceptibility to the organic solvent extracts of pomegranate peels. The pomegranate peel methanolic extract was highly efficient against *S. aureus*, MRSA, *E. coli* and *S. typhimurium* recording inhibition zones of 23.7, 21.8, 15.6 and 14.7 mm, respectively. *Staphylococcus aureus* was the most susceptible strain to pomegranate peel methanolic, acetonic and hexanic extracts with recorded zones of inhibition of 23.7, 19.5 and 17.9 mm, respectively. Our results were in consistence with that of Naziri et al. 2012 who reported that *S. aureus* revealed the highest sensitivity to the methanolic extract of pomegranate peels [33]. The same result was confirmed by Rosas-Burgos et al. 2017, who reported the high sensitivity of Gram positive bacterial strains, namely, *Bacillus subtilis, S. aureus* and *Enterococcus faecalis* to pomegranate crude extract compared to Gram negative ones [34]. The water extract of pomegranate peels revealed no antibacterial activity against MRSA, *E. coli* and *S. typhimurium* while antibacterial activity was detected against *S. aureus* strain recording zone diameter of 10.41 mm. Kupnik et al. 2021 reported that the aqueous extract of pomegranate peel showed antibacterial activity against *S. aureus* strain recording zone diameter of 11 mm [35].

In the current study, MIC values of pomegranate peel methanolic extract were 125 and 250 µg/mL against *S. aureus* and MRSA strains while MIC against *E. coli* and *S. typhimurium* was 500 µg/mL respectively. Nozohour et al. 2018 evaluated the antibacterial efficacy of pomegranate peel extracts against *Pseudomonas aeruginosa* and *S. aureus* strains recording MIC values of 12.5 and 25 mg/mL [36]. In this regard, low MIC value of pomegranate methanolic extract against the tested bacterial pathogens proved the efficacy of phytochemical extraction procedure. Difference in antibacterial bioactivity data between our findings and the previous reports may be assigned to a number of factors, including the harvesting season, plant age, the plant geographical location, extraction procedure, drying method and growth stage [37].

High efficiency of pomegranate methanolic extract against different bacterial pathogens highlights using of the extract in biofabrication of natural food preservatives avoiding side effects of synthetic food additives. A previous study recommended adding pomegranate peel extract to chicken meat products owing to the impressive extract effect in enhancing the meat shelf life and its efficient antimicrobial effect against *S. aureus* [38].

Contrastingly, the acetonic fraction of pomegranate peels showed the highest antiradical efficacy, followed by methanolic and hexanic fractions recording radical scavenging percentages of 86.9, 79.4 and 68.2%. The antiradical activity of pomegranate peel solvent extracts was assessed by adding the purple colored solution named 2,2′-diphenyl-1- picrylhydrazyl radical (DPPH) to the pomegranate extracts and observing the DPPH decolorization from purple to yellow color at 517 nm [39]. The antiradical effect of the pomegranate extracts is directly correlated to the decolorization degree in color of DPPH solution. Hence, the significant decrease in absorbance proved the potential antiradical efficiency of the tested extracts [40].

The observed high antiradical effect of pomegranate methanolic and acetonic extract may be allocated to their constituents of polyphenolic compounds as Gallic and Ellagic acids [41]. Natural polyphenolic compounds in the pomegranate extracts display their antiradical effect because of the hydroxyl groups in the phenolic aromatic rings [42]. Owing to their redox properties, these polyphenols display their antioxidant activity through neutralization of free radicals, decomposing peroxides and acting as quenching agents against singlet and triplet O_2_ [43]. The findings of the present study proved the impressive effect of pomegranate extracts as a safe and natural antioxidant against free radicals avoiding the detrimental impact of these radicals on individual health.

The methanolic extract of pomegranate peels recorded the highest level of polyphenolic compounds. Our results were in accordance with that of Rosas-Burgos et al. 2016 who attributed the high antimicrobial activity of pomegranate peel methanolic extract to its high phenolic content of 263 mg/g [34].

The acetonic, methanolic and hexanic extracts of pomegranate peels were highly efficient against MCF7 cancer cells recording IC_50_ of 8.15, 11.14 and 15.07 µg/mL. A previous report demonstrated the antiproliferative effect of the ethanolic extract of pomegranate peel against MCF7 breast cancer cells and HCT116 colon cancer cells recording IC_50_ values of 40 and 120 µg/mL, respectively. The study also displayed that the anti-proliferative efficiency of pomegranate peels against cancer cell lines may be assigned to their constituents of polyphenolic compounds [44]. In the current study, the methanolic extract of pomegranate peel was highly selective against the transformed cells (MCF7 cancer cells) and this result was in accordance with that of previous studies, which reported that the pomegranate peel extracts selectively inhibited the proliferation of lung and prostate cancer cells with no observed toxicity to normal cells [45,46]. The current study confirmed the anticarcinogenic effect of pomegranate extracts supporting the utilizing of these extracts as antiproliferative adjuvant therapy avoiding the harmful impact of chemotherapeutic agents.

The pomegranate peel methanolic extract, which revealed the highest antimicrobial efficiency, was composed of 5-Hydroxymethylfurfural as a main active component with relative percentage of 37.55%, respectively. Our results were consistent with that of Hanafy et al. 2021 who proved that 5-Hydroxymethylfurfural was the most frequent phytoactive compound in pomegranate peels ethanolic and methanolic extracts recording percentages of 48.34 and 65.78% respectively [47]. GC–MS analysis of pomegranate hexanic extract showed that Hexasiloxan, Aminopropionic acid and Dicholoroacetamide were the main phytoactive components with relative percentages of 38.28, 19.46 and 11.23%, respectively. A previous study reported that Aminopropionic acid and Alanine were the main phytochemical ingredients of pomegranate peel hexanic extract with relative percentages of 20.56 and 48.12% respectively [48]. Polyphenolic and tannin content of pomegranate extracts were suggested to possess antibacterial activity because of their capability to cause cell membrane leakage through protein precipitation resulting in cell lysis and consequently cell death [49]. The acetonic extract of pomegranate peels reported the highest antiproliferative efficiency, while the methanolic extract exerted the highest antimicrobial activity. The potential antibacterial activity of the methanolic extract of pomegranate peel may be assigned to the presence of many active phytochemicals, which have been reported to possess antimicrobial activity, such as Glycerin, Furfural, Palm tic acid, 5-Hydroxymethylfurfural and Octadecanoic acid [50,51,52,53,54]. Furthermore, the antimicrobial efficiency of pomegranate peel extracts may be due to the synergistic effect of different active phytochemicals [55]. Additionally, a high polyphenolic content of pomegranate peels contributes to the potent antimicrobial efficiency of pomegranate peels owing to their interaction with the proteins of bacterial cell membrane and/or protein sulfhydryl groups resulting in precipitation of membrane proteins, suppression of enzymes as glycosyltransferases and finally cell death induction [56]. On the other hand, the following phytochemicals of pomegranate peels detected by GC–MS analysis were reported previously to possess potential antioxidant and antiproliferative activities: Furfural, 5-hydroxymethylfurfural, 2-Furancarboxaldehyde, 5-methyl, 4H-Pyran-4-one, 3,5-dihydroxy-2-methyl, γ-Sitosterol, Hexadecanoic acid, methyl ester and Octadecanoic acid [57,58,59,60].

HPLC analysis of pomegranate peel methanolic extract revealed the presence of many polyphenolic compounds, such as cinnamic acid, gallic acid and punicalagin. Regarding bioactivity of pomegranate peel extracts, gallic acid detected by HPLC was reported to possess antiproliferative activity against human colon adenocarcinoma cells recording IC_50_ of 45.7 µg/mL [61]. Furthermore, the polyphenolic compound, punicalagin, was reported to exhibit strong antiproliferative efficiency against cervical, breast and lung cancer cell lines [62]. The main phenolic compound detected in methanolic extract of pomegranate peel was found to be cinnamic acid and this result was in agreement with that of El-Hamamsy and El-khamissi 2020 [63]. In this regard, cinnamic acid was recommended as an adjuvant antitumor agent with cisplatin chemotherapeutic drug owing to the potential antioxidant and antiproliferative activity of cinnamic acid natural agent [64]. The methanolic extract of pomegranate peel exhibited the highest antibacterial activity against the tested bacterial strains. Regarding the antimicrobial efficiency, p-coumaric acid detected in pomegranate peel methanolic extract was previously reported to possess antibacterial efficiency [65]. Furthermore, punicalagin exhibited antibacterial activity against *S. aureus* recording MIC of 0.25 mg/mL [66]. Moreover, gallic acid (GA) exhibited antibacterial and antibiofilm activity against *Shigella flexneri* strain recording MIC and MBC values of 2 mg/mL and 8 mg/mL, respectively [67]. Dey et al. 2014 reported that quercetin, which is one of the main polyphenolic compounds of pomegranate peels, exhibited antibacterial activity against *Klebsiella pneumoniae* recording MIC values of 56 μg/mL [68].

## 4. Materials and Methods

### 4.1. Preparation of Plant Extracts

Pomegranate fruits were purchased from the local market of Riyadh, Saudi Arabia. The collected plant material was identified by the herbarium of the Botany and Microbiology Department, King Saud University. The plant specimen was deposited in the herbarium with voucher number of (KSU_14702). The pomegranate peel extracts were prepared using three different organic solvents (acetone, methanol and n-hexane) possessing different polarities to ensure extraction of all active ingredients. Briefly, disinfection of pomegranate peels was performed using 0.5% sodium hypochlorite (NaOCl) then the peels were washed three successive times using sterile distilled water and finally left in shade for complete dryness. To attain a macerated powder of the pomegranate peels, the dried peels were homogenized using a mechanical mortar. Fifty grams of the peels powder were submerged into four 500 mL Erlenmeyer flasks containing 200 mL of the four different organic solvents. The extraction was carried out over a magnetic stirrer for 48 h at 25 °C and subsequently the extracts were centrifuged for 10 min at 9000 rpm for residues removal. Finally, the supernatants were filtered through Whatman filter paper no. 1 to obtain clear filtrates. The filtrates were evaporated and concentrated using a rotatory evaporator then kept in fridge at 4 °C till use. The extraction procedure of the aqueous extract was performed by submerging 50 g of peels powder in 200 mL of sterile distilled water at the room temperature. After, the extract was centrifuged at 5000 rpm for 10 min at 5 °C to remove the extract residues and finally membrane sterilization was performed using millipore filter (0.45 μm). Further dilutions of pomegranate peel water extract were made in sterile phosphate buffer saline (PBS). The extracts yield was detected according to the subsequent formula:

Extract yield = (A/B) × 100;

where A is the extract residue weight and B is raw sample weight.

### 4.2. Preparation of Microbial Suspension

Four bacterial strains namely, *S. aureus* (ATCC 29213), MRSA (ATCC 33592), *E. coli* (ATCC 25922) and *S. typhimurium* (ATCC 39183) were assayed for their sensitivity to *Punica granatum* L. (pomegranate) peel extracts. The bacterial strains were subcultured onto Mueller–Hinton broth (MHB) and incubated over a rotatory shaker at 37 °C for 24 h to obtain fresh inoculums. The turbidity of the bacterial suspension was adjusted using 0.5 McFarland standards to obtain a concentration of 10^8^ cells/mL.

### 4.3. Antibacterial Susceptibility Testing

Agar disk diffusion method was used to examine susceptibility of bacterial pathogens to different pomegranate peel extracts [69]. 1 mL of the bacterial suspension was pipetted in sterile Petri dishes then cooked Mueller–Hinton agar (MHA) medium was added and mixed well. Sterile filter paper disks (8 mm in diameter) were saturated with different pomegranate peel extracts (10 mg/disk) and positioned over the seeded layer. Chloramphenicol (30 µg) disks were applicable as (+) controls and filter paper disks, loaded with the solvents only without the extract, were applicable as (−) controls. The inhibition zone diameters were measured using Vernier caliper.

### 4.4. Determination of Minimum Inhibitory Concentration

The minimum inhibitory concentration was examined for the pomegranate peel methanolic extract as it displayed the highest antimicrobial efficiency. The broth microdilution method was utilized to detect MIC of the pomegranate peel methanolic extract using 96 well microtitre plates as described in an earlier study [70]. The tested bacterial microorganisms were cultured in Mueller–Hinton broth (MHB) and then 20 µL of the microbial suspension was pipetted into the wells containing different concentrations of the methanolic extract (0.0625–10.0 mg/mL). Positive control wells were prepared by adding 100 µL of chloramphenicol at a concentration of 30 µg/mL to the wells containing the bacterial inoculum. Negative controls were prepared by pipetting the bacterial inoculum without any extracts into the wells. Finally, incubation of the plates was done using overnight at 37 °C and read with ELISA microplate reader (Model 680, Bio-Rad Laboratories, Inc., Hercules, CA, USA). The least concentration of the pomegranate methanolic extract that exhibited no visible bacterial growth after 24 h incubation in broth microdilution assay was regarded as MIC. The experiments were carried out in triplicates.

### 4.5. Determination of Minimum Bactericidal Concentration

Minimum bactericidal concentration is explained as the least concentration of the pomegranate peel extract showing bactericidal activity. After 24 h incubation, 100 µL from the well of the micro-broth assay plates was cultured onto MHA plates then the plates were further incubated at 37 °C for 24 h. The minimum concentration of extracts exhibiting no visible bacterial growth was recorded as MBC.

### 4.6. Antioxidant Activity

The antiradical efficiency of pomegranate peel extracts was assessed using DPPH (2,2-diphenyl-1-picrylhydrazyl) assay as stated by Popovici et al. 2021 [71]. Briefly, the crude pomegranate peel extracts were dissolved in methanol for the preparation of stock solution (1 mg/mL). 2 mL of DPPH solution (0.1 mM) was prepared in absolute methanol then mixed well with pomegranate extracts in the measuring cuvettes. The mixtures were incubated in dark at 24 °C for 30 min to confirm the reaction occurrence. The mixture absorbance was measured using ultraviolet (UV)-1800 spectrophotometer at 517 nm against equal amount of methanol and DPPH as a blank. Ascorbic acid was used as a positive control. The percentage of DPPH scavenging was estimated according to the following equation:

% DPPH scavenging = [(A − B)/A] × 100,

where A is the absorbance of the control and B is the sample absorbance

### 4.7. Cytotoxicity Assay (MTT)

The MCF-7 breast cancer cells (ATCC HTB-22) as well as normal human fibroblasts WI38 (ATCC CCL-75) were supplemented from the Zoology department, College of Science, King Saud University, Saudi Arabia. The antiproliferative activity of different pomegranate peel extracts against MCF-7 cancer cells and normal human fibroblasts (WI38) were appraised using MTT assay. The cell lines were sub-cultured in the minimal essential medium (Sigma-Aldrich, St. Louis, MO, USA) enriched with 0.1% gentamicin as antibacterial agent (Virbac, Carros, France) and 5% fetal calf serum (Adcock-Ingram, Bryanston, South Africa) then the cells were incubated in a 5% CO_2_ incubator at 37 °C. The cancer cells were pipetted onto 96-well plates and kept overnight in a 5% CO_2_ incubator at 37 °C to allow cells adherence to the plate. The crude pomegranate peel extracts were suspended in methanol solvent to attain a final concentration of 10 mg/mL, and then different dilutions ranging from 0.0065 to 1 mg/mL were prepared. The cells were treated with the prepared concentrations of the pomegranate peel extracts. The supernatant was disposed after 48 h treatment and the developing solution (MTT) was added to the wells at a concentration of 5 mg/mL for the aim of formazan crystals formation. The 96-well plates were then incubated at 37 °C for 4 h and supernatants were discarded. Finally, the formed formazan crystals were stabilized by adding 50 μL of dimethyl sulfoxide (DMSO) to the wells. The absorbance of the soluble formazan was estimated using a microplate (ELISA) reader at a wavelength of 570 nm. Methanol was used as a negative control (background absorbance) while Toxol/Paclitaxel, chemotherapeutic anticancer drug, was used as a positive control [72]. The absorbance corresponding to the concentration inducing a 50% inhibition of cell viability (IC_50_) was calculated [73]. Cell viability percentage was calculated according to the following formula:

$$\%\;\mathrm{cell}\;\mathrm{viability}=\frac{\mathrm{Absorbance}\;\mathrm{of}\;\mathrm{treated}\;\mathrm{cells}-\mathrm{background}\;\mathrm{absorbance}\;}{\mathrm{Absorbance}\;\mathrm{of}\;\mathrm{untreated}\;\mathrm{cells}-\mathrm{background}\;\mathrm{absorbance}}$$

The selectivity index (SI) was evaluated as the ratio between IC_50_ values of normal and tumor cells treated with pomegranate peel extracts. The SI was estimated according to the following equation;

SI = IC_50_ (normal human fibroblasts (WI38))/IC_50_ (MCF-7 breast cancer cell line)

When the SI value is higher than 2, it is considered that pomegranate peel extract is highly selective for transformed cells compared to normal cell line [44].

### 4.8. In Vitro Hemolytic Activity

The in vitro hemolytic activity of different pomegranate peel extracts was evaluated using the methodology described by Riaz et al., 2012 [74]. The crude pomegranate peel extracts were diluted in 10% DMSO to attain a final concentration of 10 mg/mL. Twenty µL of pomegranate peel extracts were added to sterile Eppendorfs containing 180 µL of diluted blood cell suspension. RBCs count was adjusted using Hemocytometer to attain a cell count of 7 × 10^8^ cell/mL. Incubation of samples was performed for 35 min at 37 °C then agitated for 10 min and finally incubated for 5 min on ice. Centrifugation of the samples was performed 1000× *g* for 10 min to discard the intact erythrocytes. The supernatants (100 µL) were collected and diluted with 900 µL of chilled phosphate buffer saline (PBS). 200 µL of the mixture was pipetted into 96 well plates. Triton X-100 was used as a positive control showing 100% lysis while PBS buffer was used as a negative control demonstrating no hemolytic activity. The absorbance was measured at 576 nm using a microplate (ELISA) reader. The lysis percentage was measured by comparing absorbance of Triton X-100 and the samples. The percentage of hemolysis was measured according to the following formula:

$$\%\;\mathrm{hemolysis}=\frac{O.D_{S}-\;O.D_{B}}{O.D_{p}}$$

where O.D_S_ is the absorbance of the sample, O.D_B_ is the absorbance of blank (negative control) and O.D_p_ is the absorbance of positive control.

### 4.9. Determination of Total Phenolic Content

The total polyphenolic content was detected using the Folin–Ciocalteu reagent as previously described by Kupnik et al. 2021 [35]. The results were presented as gram of gallic acid equivalents (GAE) per gram of pomegranate peel extract.

### 4.10. Gas Chromatography–Mass Spectrometry

Chemical analysis of the active ingredients of pomegranate peel extracts was performed using GC–MS. The phytochemical investigation was performed using the GC–MS Thermo Trace GC Ultra/TSQ Quantum GC equipped with TR5-MS capillary column, (0.25 μm film thickness × 0.25 mm in diameter × 30 m in length). The analytical conditions were set as follows: pure helium (99.99%) as an inert carrier gas with a flow rate of 1 mL/min, the oven was set to a ramp rate of 6 °C/min to raise the temperature up to 200 °C, injector and detector temperatures were adjusted at 250 °C, the injected volume was 1 μL with split ratio of 1:50. The conditions for spectral mass detection were adjusted as follows: mass range from *m*/*z*, 40–400 amu; electron multiplier energy 2000 V; high ionization potential 70 eV; electron multiplier energy 2000 V. The bioactive components of the pomegranate peel extracts were distinguished by matching GC–MS results with the retention time and spectral database of the NIST library [75].

### 4.11. Determination of Phenolic Constituents Using HPLC

Phenolic constituents of different pomegranate peel extracts were performed using HPLC apparatus consisting of E-Chrom Tech Model LC 1620 which is a liquid chromatography system fitted with a UV detector at wavelength of 280 nm. The chemical analysis was conducted using Column C 18: Shodex C18-120-5 4 E with size of (250 × 4.6 mm), the flow rate of 1 mL per minute, Pump: P 1620A Pump, the eluents used were methanol: water: tetrahydrofuran: acetic acid with corresponding proportions of (23:75:1:1), respectively; the software used was PA Station 2015 ChemStation Version 2.0.

### 4.12. Statistical Analysis

Data were statistically analyzed with GraphPad Prism 5.0 (GraphPad Software, Inc., La Jolla, CA, USA) using one-way analysis of variance and Tukey’s test. All experiments were carried out in triplicates and the data were expressed as a mean of triplicates ± standard error.

## 5. Conclusions

The methanolic extract of pomegranate peels showed the highest antibacterial efficiency against the tested bacterial strains while the acetonic extract recorded the highest antioxidant and antiproliferative activity. Moreover, the different extracts of pomegranate peels exhibited no hemolytic activity ensuring the biosafety of these extracts. Accordingly, the impressive antimicrobial efficiency of the extracts of pomegranate peels against tested food spoilage pathogens confirmed the potential utilization of pomegranate extracts as safe food preservatives evading the harmful complications of synthetic food additives. Furthermore, the extracts also revealed a high antiradical activity against DPPH radical encouraging utilization of these extracts in fabrication of safe and natural antioxidants, thereby avoiding the possible toxic effects of the synthetic antioxidants. Furthermore, the extracts could be applicable as a promising anticarcinogenic adjuvant therapy because of their potent activity against MCF7 cancer cells.

## Figures and Tables

**Table 1:** Minimum inhibitory concentration and minimum bactericidal concentration of the methanolic extract of pomegranate peels.

Bacterial Strains	Methanolic Extract of Pomegranate Peels (mg/mL)	
	MIC	MBC
*S. aureus*	0.125	0.250
MRSA	0.250	0.500
*E. coli*	0.500	1.000
*S. typhimurium*	0.500	2.000

**Table 2:** GC–MS of the methanolic extract of pomegranate peels.

Compounds	Chemical Formula	Mol. Weight	RT	% of Total
Glycerin	C_3_H_8_O_3_	92.09	5.623	7.74
Furfural	C_5_H_4_O_2_	96.08	5.717	14.62
Cyclobutylamine	C_4_H_7_NH_2_	71.12	7.615	1.58
4H-Pyran-4-one, 3,5-dihydroxy-2-methyl	C_6_H_8_O_4_	144.12	9.487	1.14
Pyrazole[4,5-b]imidazole, 1-formyl-3-ethyl-6-β-d-ribofuranosyl	C_12_H_16_N_4_O_5_	296.28	10.672	2.39
L-Glucose	C_6_H_12_O_6_	180.16	14.926	1.07
Palmitic acid	C_16_H_32_O_2_	256.42	15.236	1.18
5-Hydroxymethylfurfural	C_6_H_6_O_3_	126.11	17.374	37.55
Heptasiloxane, hexadecamethyl-	C_6_H_16_O_2_Si	148.28	19.066	3.14
Octadecanoic acid	C_18_H_36_O_2_	284.48	22.165	16.89
γ-Sitosterol	C_29_H_50_O	414.71	25.982	9.23
Lanosterol	C_30_H_50_O	426.72	31.365	1.82
Cycloartenol acetate	C_32_H_52_O_2_	468.76	34.182	1.64

**Table 3:** GC–MS of the acetonic extract of pomegranate peels.

Compounds	Chemical Formula	Mol. Weight	RT	% of Total
Furfural	C_5_H_4_O_2_	96.08	4.793	11.29
2-ethyl-1,3-dimethyl-benzene	C_10_H_14_	134.21	4.927	4.25
Hexadecanoic acid, methyl ester	C_17_H_34_O_2_	270.45	5.783	1.89
α-Cubebene	C_15_H_24_	204.35	7.187	1.06
4H-Pyran-4-one, 3,5-dihydroxy-2-methyl,	C_6_H_8_O_4_	144.12	9.163	3.67
2,5-Furandione, 3-methyl	C_5_H_4_O_3_	112.08	9.817	7.12
2-Furancarboxaldehyde, 5-methyl	C_6_H_6_O_2_	110.11	11.284	9.58
D-Arabinose	C_5_H_10_O_5_	150.13	12.456	5.78
4-Methyl itaconate	C_6_H_8_O_4_	144.12	15.358	2.45
5-hydroxymethylfurfural	C_6_H_6_O_3_	126.11	17.236	28.84
n-Hexadecanoic acid	C_16_H_32_O_2_	256.43	17.897	6.85
N-phenyl-2-naphthalenamine	C_16_H_13_N	219.28	19.681	8.47
Squalene	C_30_H_50_	410.70	21.578	4.29
Eicosane	C_20_H_42_	282.55	24.185	1.56
Lanosterol	C_30_H_50_O	426.71	29.257	2.89

**Table 4:** GC–MS of the hexanic extract of pomegranate peels.

Compounds	Chemical Formula	Mol. Weight	RT	% of Total
Aminopropionic acid	C_3_H_7_NO_2_	89.09	3.634	19.46
Dicholoroacetamide	C_2_H_3_Cl_2_NO	127.95	4.146	11.23
Benzeneacetic acid	C_8_H_8_O_2_	136.15	4.935	1.28
4H-Pyran-4-one, 2,3-dihydro-3,5-dihydroxy-6-methyl-	C_6_H_8_O_4_	144.12	6.357	1.98
2,6-Di-tert-butylphenol	C_14_H_22_O	206.32	8.439	8.12
Trioxsalen	C_14_H_12_O_3_	228.24	9.637	6.78
Octadecenoate	C_18_H_33_O_2_	281.50	12.842	2.78
2,6-Dimethyl-3,4-bis(trimethylsilyloxymethyl)pyridine	C_15_H_29_NO_2_Si_2_	311.57	16.751	5.23
Octadecanoic acid, 2-propenyl ester	C_21_H_40_O_2_	324.50	18.265	4.85
Hexasiloxan, tetradecamethy	C_14_H_42_O_5_Si_6_	458.99	26.483	38.28

**Table 5:** Different phenolic compounds identified in methanolic extracts of pomegranate peels by HPLC.

Compounds	Chemical Formula	Retention Time (min.)	Concentration (mg/mL)
Protocatechuic acid	C_7_H_6_O_4_	7.98	10.78
p-coumaric acid	C_9_H_8_O_3_	12.32	19.85
Punicalagin	C_48_H_28_O_30_	13.62	9.12
Gallic acid	C_7_H_6_O_5_	14.94	7.89
Cinnamic acid	C_9_H_8_O_2_	16.21	31.69
Quercetin	C_15_H_10_O_7_	20.32	20.22

## Data Availability

The datasets used and/or analyzed during the current study are available from the corresponding author on reasonable request.
